# Essential oils and Lactobacillus metabolites as alternative antibiofilm agents against foodborne bacteria and molecular analysis of biofilm regulatory genes

**DOI:** 10.1038/s41598-025-89998-8

**Published:** 2025-03-04

**Authors:** Hala A. Ammar, Ragda Samy, Fifi M. Reda, Wesam A. Hassanein

**Affiliations:** Botany and Microbiology Department, Faculty of Science, Zagazig University, Zagazig, Egypt

**Keywords:** Biofilm formation, *B. cereus*, Essential oils, Antibiofilm, Molecular analysis, Biotechnology, Microbiology, Molecular biology

## Abstract

The formation of biofilm by foodborne pathogens increases the risk of foodborne diseases, resulting in major health risks. Research on strategies for eliminating biofilm formation by foodborne pathogens is urgently needed. Therefore, the objective of this study was to construct a new technique for controlling foodborne bacteria and inhibiting the biosynthesis of biofilm via using natural products. The essential orange oil (EOO) and cell-free filtrate of *Lactobacillus pentosus* RS2 were used as antibacterial and antibiofilm agents against *B. cereus* RS1, the strongest biofilm-forming strain*.* The mixture of cell-free filtrate (CFF) and EOO (CFF/EOO) was the best antibiofilm agent under all tested conditions. The minimal inhibitory concentration (MIC) test revealed that 400 μl ml^−1^ CFF and 16 μl ml^−1^ EOO completely inhibited the growth of *B. cereus*. The treatment of three commercial surfaces with CFF/EOO resulted in a high reduction in biofilm synthesis, with adhesion percentages of 33.3, 36.3, and 40.8% on stainless steel, aluminum foil, and aluminum, respectively. The aluminum surface had the greatest adhesion with *B. cereus* RS1 among the three tested surfaces. These results were confirmed by expression analysis of three essential coding genes, *sinR*, *calY*, and *spo0A,* participating in biofilm formation in *B. cereus*. The biofilm-negative regulator gene *sinR* was overexpressed, whereas the biofilm-positive regulator genes *calY* and *spo0A* were down-expressed in *B. cereus* RS1 after treatment with antibiofilm agents, compared with those in the untreated sample. This study revealed that CFF/EOO was more effective at activating *sinR* (2.099 ± 0.167-fold increase) and suppressing *calY* and *spo0A* (0.314 ± 0.058 and0.238 ± 0.04-fold decrease, respectively) compared to control. This result confirmed the biochemical estimation of biofilm formation in *B. cereus* after treatment with all the experimental agents. The EOO and CFF of *L. pentosus* RS2 can be used as strong antibacterial and antibiofilm agents against foodborne bacteria. These products reduced the biofilm formation on trade surfaces affecting the expression of three essential biofilm regulatory genes. This study considered novel research concerning the potential antibiofilm activity of EOO combined with CFF of *L. pentosus* and the molecular analysis of genes regulating biofilm production under stress of CFF/EOO.

## Introduction

Foodborne disease is a major public health issue worldwide. This problem results from the ingestion of food contaminated with spoiling bacteria^1^. Many foodborne bacteria can make planktonic aggregation, adhering to each other, and encapsulate in structural colonies^2^. Food pathogens such as* Bacillus cereus*, *Escherichia coli*, and *Listeria monocytogenes* broadly contaminate food reservoirs, demonstrating considerable impedance to food safety and participation in foodborne diseases^3^. *B. cereus*, a widespread food spoilage pathogen, poses a considerable threat to food security owing to its strong flexibility under unfavorable surroundings such as ultraviolet radiation and raised temperatures^4^. *B. cereus* can cause symptoms of food poisoning, such as vomiting and diarrhea^5^. Recently, food toxicity caused by *B. cereus* has proliferated worldwide^6^. This risk is due to contaminated foods, including meat and dairy output, which are the main origin of contamination with *B. cereus*^7^. *B. cereus* can attach to alive or solid surfaces, producing extracellular polysaccharides, proteins, water, and nucleic acids, known as a biofilm. Forming biofilm by pathogenic bacteria displays a secured model of bacterial growth that permits the cells to remain alive in abnormal habitats, facilitating their colonization of recent areas*.* This layer protects microbial cells from antibiotic effects, dynamic environments, and disinfectants^8,9^. Compared with free-living bacteria, bacteria within biofilms have different physiological functions. This leads to an extension in the antibiotic tolerance of ten to thousand times^10^. It is critical to impede biofilm synthesis during food processing to guarantee both generic health and food safety. Therefore, a strong alternative agent is necessary to reduce this production by creating suitable medicines against biofilm synthesizing.

Probiotic bacteria can inhibit several food pathogens. They effectively hinder gastrointestinal-related diseases by reducing lactose intolerance^11^, preventing infection of the intestinal tract^12^, facilitating nutrient absorption and reducing allergic reactions^13^. They also have a potent antimicrobial agent against opportunistic pathogens such as the multidrug-resistant *Pseudomonas aeruginosa*^14^. Probiotic microorganisms produce bactericidal metabolites such as hydrogen peroxide, lactic acid, proteases, and lysozymes. These substances can cause enzyme inhibition and membrane disturbance of different multidrug-resistant bacteria. The mechanism of action of bacteriocins produced by LAB can replace antibiotics and prevent bacterial resistance. Ahmed and Al-Awadi^15^ reported that the probiotic bacteria, *Enterococcus faecium*, has a potential antimicrobial activity against multidrug-resistant bacteria. Previous studies have shown that *Lactobacillus plantarum* and *Lactobacillus fermentum* can inhibit biofilm biosynthesis in *Staphylococcus aureus*^16^. Potential antibiofilm efficiency of *L. plantarum* was previously investigated against multidrug-resistant pathogenic *E. coli*^17^. Additionally, natural products such as essential oils (EOs) can be used as antibiofilm and antimicrobial agents against pathogenic microorganisms. EOS could be extracted from various parts of aromatic plants, such as bark, leaves, flowers, seeds, and stems^18^. These compounds include different bioactive materials that significantly influence human and animal health. EOs have considerable therapeutic characteristics, such as antiseptic, antioxidant, and antibacterial activities^19,20^. They were previously applied as antimicrobial and antibiofilm agents against *S. aureus* strains^21^. For this reason, some commercial EOs were also tested as antimicrobial and antibiofilm agents in this study.

In *Bacillus subtilis*, biofilm synthesis regulatory networks have been discovered^22,23^. However, the regulatory mechanism concerning biofilm production in *B. cereus* is deficiently understood. Therefore, some problems associated with *B. cereus* biofilm synthesis, such as biosynthesizing genes, elucidation of signaling pathways, and quorum sensing, have become focal points in different studies^24–26^. The sin operon comprises two significant gene products, SinR and SinI. SinR is a principal transcriptional regulator and is considered a master regulator for biofilm biosynthesis. SinR is a multi-regulator of various late-growth operations and a tetrameric DNA-binding protein. Its activity is downregulated through the formation of a SinR: SinI complex. The binding of DNA with SinR results in the repression of the operons involved in the synthesis of the tapA-sipW-tasA, proteins epsA-O, and biofilm matrix polysaccharides^27,28^. When SinI complexes with SinR, the SinR tetramer is interrupted and cannot bind DNA. SinR is an antagonist of SinI, and its expression is increased by environmental cues that promote biofilm formation. Thus, the expression of SinR suppresses the transcription of biofilm coding genes. Furthermore, SinI-SinR and Spo0A-AbrB are the principal genetic networks regulating biofilm synthesis in *B. subtilis*^27,29^. The *spo0A* homologous gene of *B. subtilis* was identified as a key biofilm regulator in *B. cereus*^30^. Recently, Zhang et al.^31^ explained the function of the CalY protein and SinR /SinI system in regulating biofilm biosynthesis in *B. cereus*. Therefore, we aimed in this study to investigate a novel potential antibiofilm agent and to determine the expression of the biofilm regulatory genes *calY, sinR*, and *spo0A*, under the effects of the experimental antibiofilm agents CFF, EOO, and CFF/EOO. The investigation of the antibiofilm action of *L. pentosus* and EOO against *B. cereus* and expression analysis of biofilm-coding genes under their effects are considered a novel study.

## Materials and methods

### Materials

All culture media and reagents used in our research were obtained from Sigma‒Aldrich (St. Louis, MO, USA).

### Isolation and maintenance of lactic acid bacteria and foodborne pathogens

Fifty samples of different food products including saltfish, fish, salmon, white cheese, sausage, beef, meat, chicken, and luncheon, salad water (five samples of each), collected from different Egyptian food stores and markets, were used for the isolation of foodborne bacteria. Serial dilutions (0.1 mL) of each sample were prepared and spread on the surface of suitable culture media. MacConkey agar medium was used for the isolation of Gram-negative bacteria while nutrient agar medium was used for the isolation of Gram-positive bacteria. Fifteen samples of fermented milk including white cheese, salt cheese, yogurt, five samples of saltfish, and five samples of pickles were collected from different Egyptian markets and used for isolation of LAB. The surface spread technique was used to isolate LAB on De Man Rogosa Sharpe Agar plates^32^. All the purified colonies were preserved at 4 °C on nutrient agar slants.

### Phenotypic and genotypic identification of bacterial species

Phenotypic identification was carried out according to gram stain, biochemical reactions, cell shape, colony, and morphology of the selected LAB and foodborne pathogens^33^. *L. pentosus* and the pathogenic bacterial strain *B. cereus* were additionally identified via MALDI-TOF MS via the VITEK MS technique (bioMérieux) at 57,375 hospitals, Egypt, according to Fenselau and Demirev^34^.

The genotypic identification of our strains was performed with a 16S rDNA genetic marker. The amplification of 16S rDNA was completed via the common primers 27F and 1492R, with a 1400 bp expected amplicon^35^. The PCR reaction mixture contained 1 μL of 50 mM MgCl_2_, 2.5 μL of 10 × Taq buffer, 2.5 μL of 2 mM dNTPs, 10 pmol of 27 forward and 1492 reverse primers (1 µL each), 0.2 μL of Taq Polymerase (5 U μL^− 1^, Invitrogen™) and 2 µL of template DNA (approximately 10 ng), with a total volume of 25 μL with sterilized ultrapure H_2_O. The reaction was completed using a GeneAmp PCR apparatus 9700 (Applied Biosystems™) thermocycler. The PCR reaction was adjusted as follows: 3 min of initial denaturation at 96 °C, for 35 cycles of 30 s denaturation at 94 °C, 30 s annealing at 58 °C, a 1 min extension at 72 °C, and a final extension for 10 min at 72 °C. Five microliters of each amplified mixture were separated and detected on a 1% agarose gel (*w*/*v*). The purified amplicon was sequenced.

The obtained sequences of *B. cereus* and *L. pentosus* were registered under the accession numbers OP278950 and OP278963, respectively, at the GenBank database (www.ncbi.nlm.nih.gov). The Basic Local Alignment Search Tool (BLAST) program, http://www.ncbi.nlm.nih.gov/blast, was used to determine the relation of our sequences with the homologous sequences registered in the GenBank database.

### Estimation of biofilm production

Biofilm production by the isolated bacteria was evaluated via crystal violet (CV)^36^ and the Congo red agar (CRA) assay methods^37^. The culture medium for the CRA method consisted of 37 g^−l^ brain heart infusion, 8 g^−l^ CR stain, 50 g^−l^ sucrose, and 10 g^−l^ agars. The prepared sterilized CR stain solution was inserted into autoclaved BHI agar. The culture medium was streaked with foodborne isolates and grown at 37 °C for 24 h. The pink colonies were identified as negative biofilm-forming bacteria, the dry structure colonies without crystalline were identified as weak biofilm-forming bacteria, and the dry black crystalline colonies of foodborne pathogens were identified as vigorous biofilm-producing bacteria.

In the crystal violet method, the foodborne pathogens were evaluated for biofilm formation using the microtiter plates. The wells were loaded with BHI broth, and then they were inoculated with the foodborne pathogens. The plates were incubated at 30 °C for 48 h. Then, the wells were cleaned twice with saline phosphate-buffer, pH 7.1 (1 mM KH_2_PO_4_, 0.14 M NaCl, 3 mM KCl, and 10 mM Na_2_HPO_4_). The formed biofilm was settled by methanol for 30 min, and stained with 0.1% CV. The holes were washed by PBS 3 times. Seventy percent ethanol was inserted, and the mixture remained 30 min stable, to solubilize the adhered foodborne pathogens. The optical density (OD) was detected at 595 nm via ELISA, Microplate reader, Molecular Devices, LLC, San Jose, CA, USA.

### Antimicrobial activity of EOs and LAB against foodborne bacteria

The antimicrobial activities of the CFF of the LAB isolates and six types of emulsified essential oils, namely, thyme, garlic, orange, rosemary, lavender, and lemon grass, were estimated against *B. cereus* RS1 via the well diffusion method. The CFF was sterilized using 0.22 µm Millipore filter. 10^6^ CFU^−l^ of *B. cereus* RS1 was spread on the surface of BHI agar. A 6 mm well was constructed, and each well was filled with the CFFs of LAB and EOs. The plates were incubated at 37 °C for 24 h, and the inhibition zones (mm) were estimated^38^.

### Susceptibility test of antibiotic against *B. cereus* RS1

Antibiotic susceptibility was estimated via a disc-diffusion assay according to the European Committee on Antimicrobial Susceptibility Test guidelines^39^. Seven antibiotic discs (Oxoid) containing amoxicillin (AX 25 μg), cefotaxime (CTX 30 μg), oxacillin (OX 5 μg), gentamicin (GEN 10 μg), penicillin G (PEN 10 μg), vancomycin (VA 30 μg) and tetracycline (TET 30 μg) were used to estimate the sensitivity test on Mueller Hinton agar. The sensitivity of *B. cereus* RS1 to experimental antibiotics was evaluated by calculating the inhibition area (mm).

### Antibiofilm activity of the CFF of *L. pentosus* RS2 and essential orange oil, separately and in combination, against *B. cereus* RS1

A biofilm inhibition test of the CFF of *L. pentosus* RS2, EOO, and a mixture of CFF and EOO against *B. cereus* RS1 was performed via a coincubation test according to Gudiña et al.^40^. Twenty microliters of 10^6^ CFU^-ml^ *B. cereus* RS1 were inserted into the wells with EOO (200 µl), CFF (200 µl), or 100 µl of CFF/100 µl of EOO and grown at 37 °C for 48 h. The PBS buffer (pH 7.2) was used for washing the wells and repeated 3 times. The biofilm production of *B. cereus* RS1 was calculated via an ELISA microplate reader. Untreated *B. cereus* RS1 culture was used as a control. The microbial adhesion percentage was measured according to Fracchia et al.^41^ using the following equation: %Microbial adhesion = (Ac/A0) × 100. The absorbance of the control is represented by A_0_, and the absorbance of the EOO, CFF or CFF/EOO is represented by A_C_.

### Determination of the MICs of the CFF of *L*.* pentosus* RS2, EOO, and the CFF/EOO mixture against the selected biofilm-forming bacteria

A modified turbidity test was carried out according to Schwalbe et al.^42^. Various concentrations of the CFF of* L*. *pentosus* RS2 (50–1000 µL ml^-1^) and various concentrations of essential orang oil (2–1000 µl ml^-1^) were added separately to *B. cereus* RS1 (10^6^ CFU/mL) and to 5 ml of nutrient broth. The MIC in liquid culture was calculated after 24 h of growth at 37 °C according to Kowalska-Krochmal and Dudek-Wicher^43^. Half the sub-MIC of the CFF of *L. pentosus* (100 µl ml^-1^) and 12/sub-MIC of EOO (4 µl ml^-1^) were mixed and tested against *B. cereus* RS1. The combination of 12 /sub-MICs of the CFF of *L. pentosus (*100 µl ml^-1^*)* and 12/sub-MICs of EOO (4 µl ml^-1^) were used to detect increases or decreases in the expression of biofilm-synthesizing genes.

### Anti-adherence activities of the CFF of LAB, orange oil and CFF/EOO against biofilm production by *B. cereus* RS1 on trade materials

Biofilm production was estimated on three surfaces: aluminum foil, stainless steel, and aluminum sheets. The sheets were cleaned with acetone and alcohol (Sigma‒Aldrich, USA), deposited in HCl (2 N) for 2 h, and washed by sterilized distilled water. After that, the materials were sterilized at 121 °C for 20 min. One sterilized piece from each material was inserted into a 100 mL flask for additional survey. BHI broth was inoculated by 1 ml of 106 CFU *B. cereus* RS1 in the presence of the three experimental materials. The inoculated broth was incubated for 48 h at 37 °C. After the incubation time, the materials were separated and cleaned by 1 mL PBS (pH 7.1). The antibiofilm activity of previously prepared concentrations (sub-MICs) of the CFF of *L. pentosus*, orange oil and 1/2sub MIC CFF/ٌEOO against the adherence of the selected strain to stainless steel, aluminum and aluminum foil sheets was assayed as described by Reda^44^. Biofilm formation by the tested strains on stainless steel, aluminum and aluminum foil sheets was measured via the CV assay method as described above. Additionally, after 2 days, three sheets from each surface were removed from the tube and rinsed with PBS (pH 7.1) to clean the non-attached cells. Then, the sheets were stained with crystal violet.

### Identification of biofilm coding genes in the *B. cereus* RS1 genome via the conventional PCR technique and primer design

To extract the genomic DNA, the untreated *B. cereus* RS1 was inoculated to nutrient broth culture and grown under shaking conditions for 17 h at 37–38 °C. Traditional PCR analysis of the three biofilm coding genes (*sinR, calY*, and *spo0A*) was evaluated via the designed oligonucleotide primers. PCR mixture was prepared in 20 µL using the following reagents: ten pmol of primers (1 µL each forward and revers), 2 µL template DNA (10 ng), 0.2 µL DNA HF-polymerase, 4 µL 5 × Phusion HF Green buffer, and 0.4 µL 10 mM dNTPs and completed with nuclease-free water to total volume 20 µL. PCR steps were an initial denaturation, 98 °C for 30 s, and adjusted to 35 cycles of 10 s denaturation at 98 °C, 15 s annealing at 58 °C, 20 s extension at 72 °C, and 5 min final extension at 72 °C. agarose gel electrophoresis (1.5%) in 1 × TEA buffer was used to examine the PCR products. Gel electrophoresis was used to analyze PCR products. Five µL of the DNA outcome were filled in the gel hole. The fragment size was calculated using a 100 bp DNA Ladder (QiagenA gel documentation system was used for the photographic process. The Primer 3 plus software (http://primer3plus.com/cgi-bin/dev/primer3plus.cgi) was used to design the primers of biofilm coding genes as recorded in Table 1. Conventional PCR technique was applied to detect the amplification of the corresponding fragments by the designed primers. All amplicons were recognized via gel electrophoresis technique. All primers of this experiments were got from Sigma‒Aldrich, St. Louis, MO.

**Table 1:** Oligonucleotide primer sequences of biofilm coding genes and rRNA genes.

Primers ID	Primer sequence (5’–3’)	Target gene	Annealing temperature (Tm)	Size of amplicon (bp)	References
*16 s27F*	AGA GTT TGA TCM TGG CTC AG	*16SrRNA*	57.9	1400	Heuer et al.^35^
*16 s U1492R-*	CGGT TAC CTT GTT ACG ACT T		58		
*sinR-F*	AAAAAGCTGGCGTTGCTAAA	*sinR*	60.79	350	Current study
*sinR-R*	GGAGACACCAGAGTTCATTGC		59.94		
*calY-F*	ATTTGCAGCTGGGACGTTAG	*calY*	60.27	347	Current study
*calY-R*	CCCACTCAGGAGCAAAGATG		60.79		
*Spo0A-F*	TCATTCCGATCAGCAACAAC	*Spo0A*	59.65	380	Current study
*Spo0A-R*	CACGGCTTGCTGTGGTATTA		59.75		
*dnaJ-F*	GGCAGGGGACAAGTAGGAA	*dnaJ*	61	227	Marghmaleki et al.^45^
*dnaJ-R*	CCCCTATTGCCACTTTTGCT		61		

### Expression, sequencing, and phylogenetic analyses of biofilm-regulating genes

Quantitative real-time PCR (qRT‒PCR) was carried out to evaluate the expression of biofilm coding genes in *B. cereus* RS1. The biofilm-producing strain *B. cereus* RS1 was inoculated into a biofilm-producing culture medium supplemented with CFF, EOO, or a mixture of both CFF and EOO and incubated at 37 °C for 17 h. The untreated biofilm-producing *B. cereus* RS1 was used as a control. According to the manufacturer’s instructions, the total RNA of all the samples was extracted via a QIAamp RNeasy Mini Kit (Qiagen, Germany). The expression values of the biofilm regulator gene (*sinR*) and the coding genes (*calY* and *spo0A*) were amplified via the designed primers (Table 1). Q-RT‒PCR was carried out in a total volume of 25 µL at the following reagents: 12.5 μL of 2 × QuantiTect SYBR Green PCR Master Mix (Qiagen), 0.5 μL of each primer (10 pmol μL^−1^), 1.25 μL of RT enhancer (Thermo Scientific), 2.5 μL of cDNA template, 0.25 μL of Verso Enzyme Mix (including RNase inhibitor), and 6.5 μL of H_2_O. The reaction was carried out in a Strata-Gene MX3005P real-time PCR machine. The PCR conditions were as follows: reverse transcription for 30 min at 50 °C; initial denaturation for 5 min at 94 °C; 40 cycles of secondary denaturation for 15 s at 94 °C, annealing for 1 s at 58 °C, and extension for 45 s at 72 °C; and 1 cycle of denaturation for 1 min at 94 °C, for dissociation curve analysis, annealing at 54 °C for 1 min, and finally denaturation at 94 °C for 1 min. The amplicon size was determined via agarose gel electrophoresis analysis. The *dnaJ* was used as a housekeeping gene^45^. The released data were calculated with IQ5 optical system software (Bio-Rad, Hercules, CA). The cycles threshold was determined via the baseline subtracted mode. The qualities of the amplification of the amplified genes from untreated and treated strains of *B. cereus* RS1 were evaluated. The amplification curves and CT values were estimated using Strata gene MX3005P software (Strata gene, La Jolla, CA). The CT value of the *dnaJ* was used for normalization of q-RT PCR. To calculate the change in the expression level of the biofilm coding genes, the CT values of the samples were matched to that of the untreated sample (positive control) as stated by the BΔΔCt method^46^. The PCR products of the biofilm coding genes were sequenced in both forward and reverse directions via a ready reaction Bigdye Terminator V3.1 cycle sequencing kit (Perkinelmer/Applied Biosystems; Cat. No. 4336817), using an Applied Biosystems 3130 automated DNA sequencer (ABI, 3130, Applied Biosystems, Foster City, CA). All sequences were annotated by the Basic Local Alignment Search Tool (Blast) using the NCBI database, http://blast.ncbi.nlm.nih.gov. The sequences of the biofilm-coding genes were deposited in the GenBank database. The ClustalW program in MEGA11 software was used to construct the phylogenetic trees of the released proteins and DNA sequences by multiple sequence alignments. The evolutionary pedigree was deduced by the maximum parsimony method. The distances of evolution were calculated by the p-distance algorithm and are drawn in units of the amino acid differences/site. The evolutionary test was performed via MEGA11 software^47^. The conserved parts of the annotated biofilm coding genes were examined for their similar families of protein using the conserved domain database at the website https://www.ncbi.nlm.nih.gov/cdd/.

### Statistical analysis

All experiments were performed in triplicate and the data were statistically analyzed using one-way analysis of variance (ANOVA) with post hoc pairwise comparisons adjusted by Tukey’s post-test. The result was considered statistically at *p* <0.05. All values are expressed as mean ± SD.

## Results

### Screening study for biofilm formation by foodborne pathogens and the antibacterial activity of LAB

**Fig. 1:**
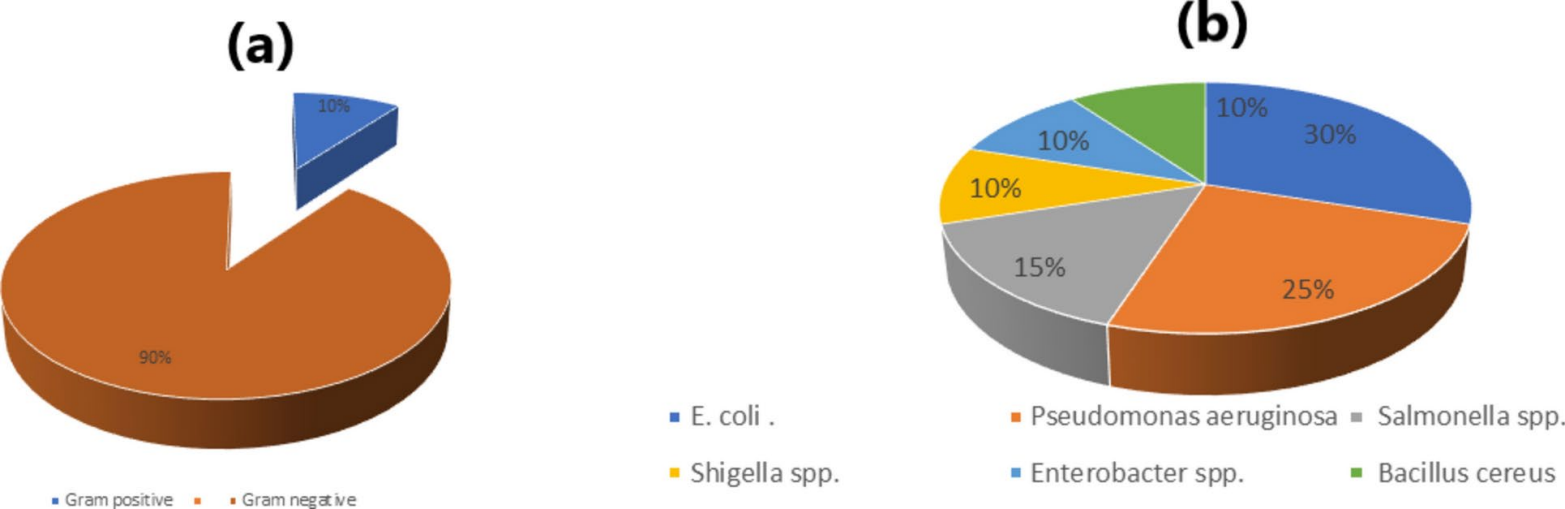
Distribution of bacterial isolates according to their Gram’s stain reaction (**a**) and prevalence of the isolated food borne pathogens in the tested food samples (**b**).

Forty foodborne pathogens, 36 Gram-negative bacteria (90%) and 4 Gram-positive bacteria (10%), were isolated from different Egyptian food samples (Fig. 1a). The number of Gram-negative bacteria was greater than the number of Gram-positive bacteria in all the tested sources. The foodborne isolates were classified into six groups: *E. coli*, *Salmonella* spp., *Shigella* spp., *Enterobacter* spp., *Pseudomonas aeruginosa* and *B. cereus.* The most common isolates were *E. coli* (30%), followed by *P. aeruginosa* (25%), *Salmonella spp*. (15%), *Shigella* spp. (10%), *Enterobacter spp*. (10%) and *B. cereus* (10%), as shown in Fig. 1b*.* All the bacterial isolates were screened for their ability to form biofilms qualitatively and quantitatively. The results revealed that all the isolates produced black colonies on CRA plates, and their ODs were high or moderate*.* The *B. cereus* isolate RS1 was the most potent biofilm-producing pathogen (OD 0.985); consequently, it was selected as an experimental species for this study. Additionally, thirty-six LAB were obtained from pickles, saltfish and fermented dairy products, and their antagonistic activities were tested against the highest biofilm-producing strain, *B. cereus*. The results showed that the greatest inhibitory effect was obtained by the LAB isolate RS2 against the *B. cereus* RS1 isolate with a diameter of 20 mm.

### Bacterial identification

**Fig. 2:**
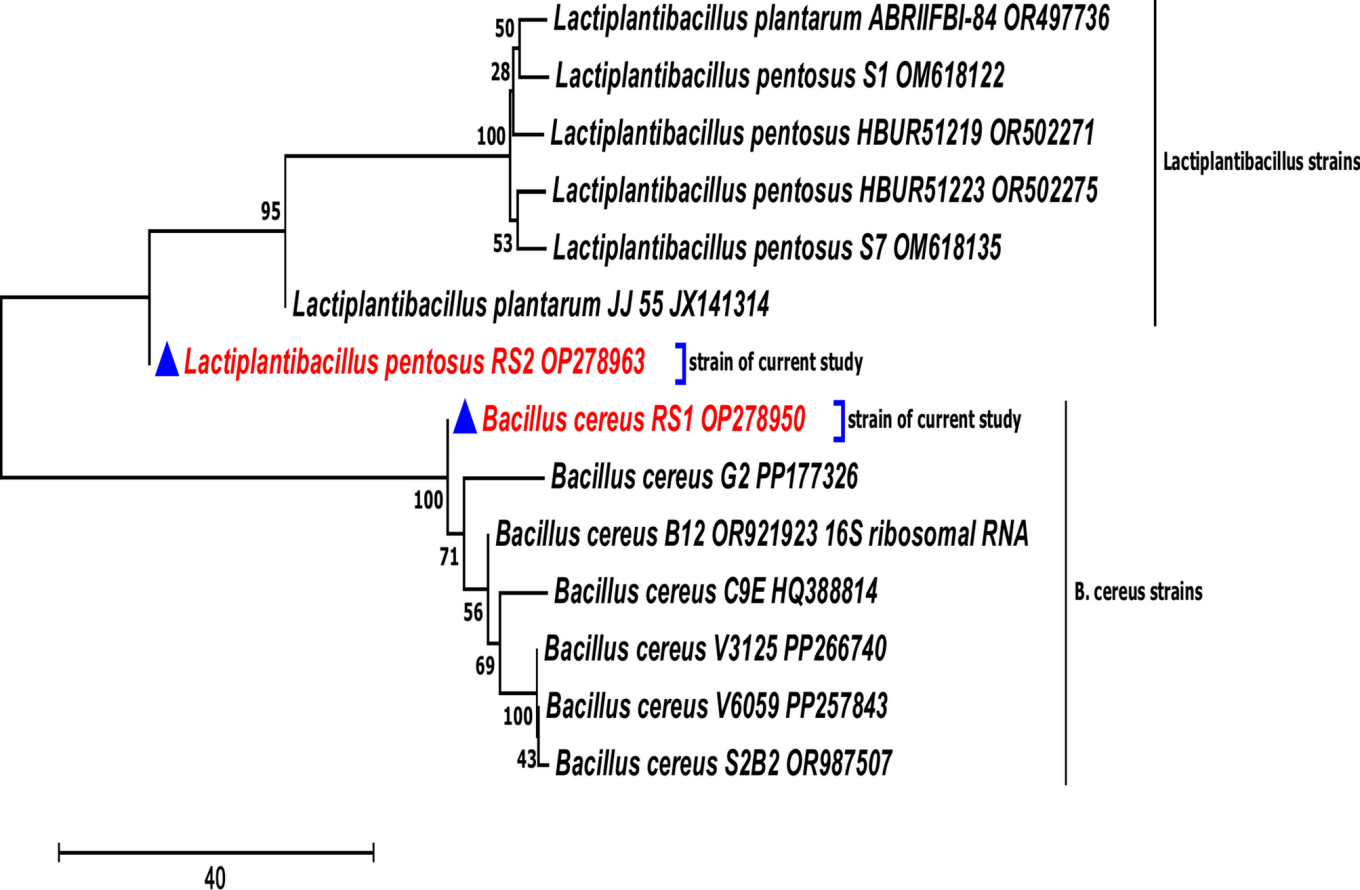
Phylogenetic analysis of 16 s rRNA of *Lactobacillus pentosus* RS2 OP278963 and *B. cereus* RS1 OP278950 with closely related sequences of bacterial strains retrieved from NCBI GenBank. Evolutionary history was inferred using the Neighbor-Joining method. The optimal tree with the sum of branch length = 170.08512370 is shown. The percentage of replicate trees in which the associated taxa clustered together in the bootstrap test (1000 replicates) are shown next to the branches. The tree is drawn to scale, with branch lengths in the same units as those of the evolutionary distances used to infer the phylogenetic tree. The evolutionary distances were computed using the number of differences method and are in the units of the number of base differences per sequence. The analysis involved 14 nucleotide sequences. All ambiguous positions were removed for each sequence pair. There were a total of 1563 positions in the final dataset. Evolutionary analyses were conducted in MEGA11.

Phenotypic analysis via microscopic examination, biochemical tests and MALDI-TOF MS by the VITEK MS technique revealed that the LAB was *L. pentosus* RS2 and the pathogenic strain was *B. cereus* RS1. This identification was confirmed by molecular analysis of ribosomal RNA using the genetic marker 16S rRNA. The partial nucleotide sequences of the amplified genes from *L. pentosus* and *B. cereus* were submitted to the GenBank database under the accession numbers OP278963 and OP278950, respectively. The phylogenetic trees shown in Fig. 2 represent the percentage of similarity between the tested strains and their related strains deposited in the GenBank database. The phylogenetic analysis revealed 99–100% similarity between *L. pentosus* RS2, OP278963, and *L. pentosus*, OM618135, *L. pentosus*, OR502275, and *L. pentosus*, OR502271. Additionally, it showed 99–100% similarity between *B. cereus* RS1*,* OP278950 and *B. cereus,* G2 PP177326, *B. cereus,* B12 OR921923, and *B. cereus,* C9E HQ388814.

### Antimicrobial activity of essential oils against *B. cereus* RS1 and antibiotic susceptibility test

**Fig. 3:**
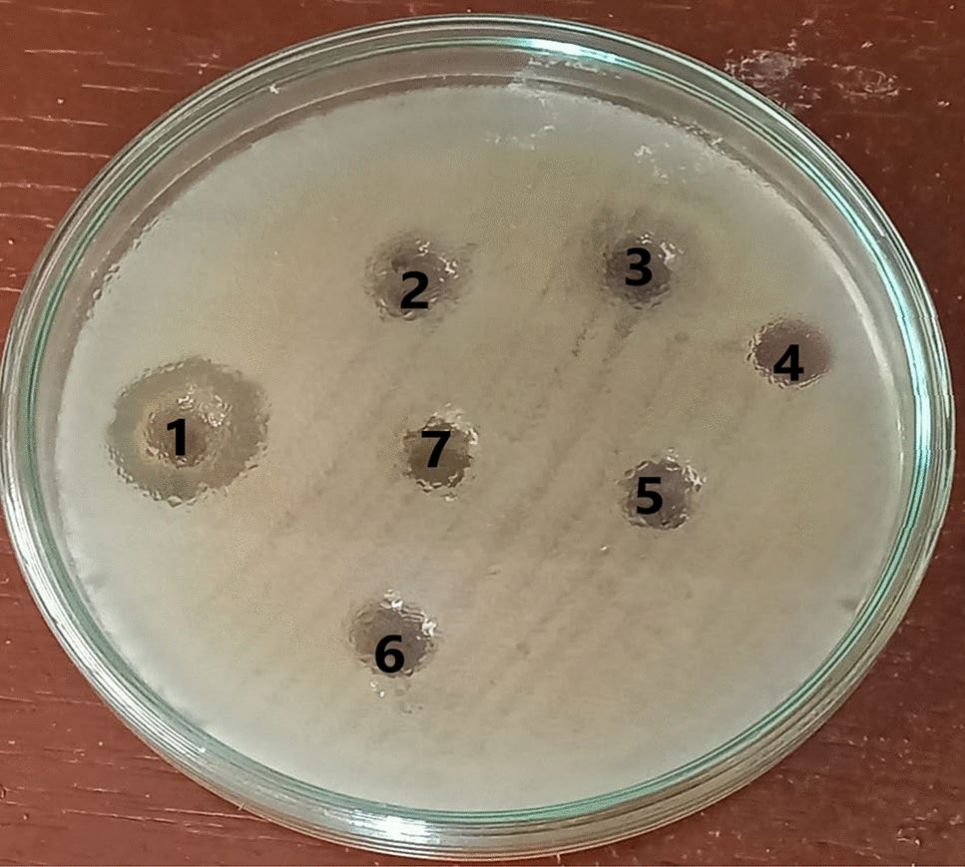
Antibacterial activity of some selected EOs including Orange (1), Rosemary (2), Lemongrass (3), Garlic (4), Lavender (5), and Thyme (6) against *B. cereus* RS1.

**Fig. 4:**
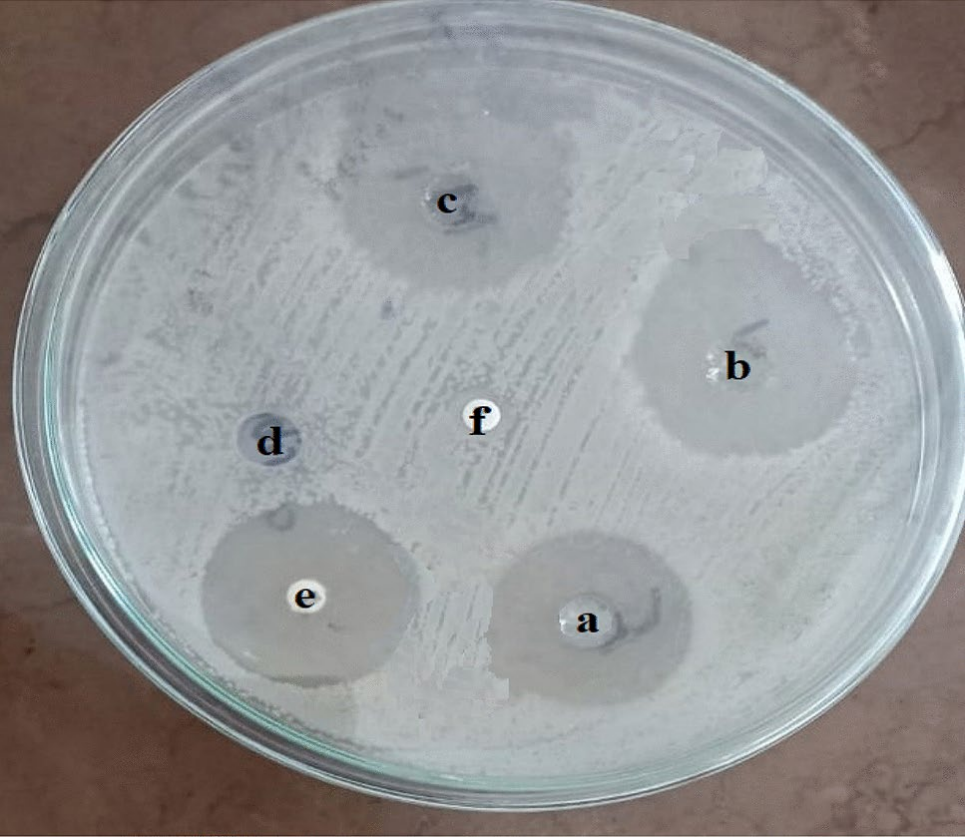
Antibacterial activity of EOO (**a**), CFF (**b**), and EOO/CFF (**c**) in the presence of water and Oxacillin as negative controls (**d**, **f**, respectively) and tetracycline as a positive control (**e**).

**Fig. 5:**
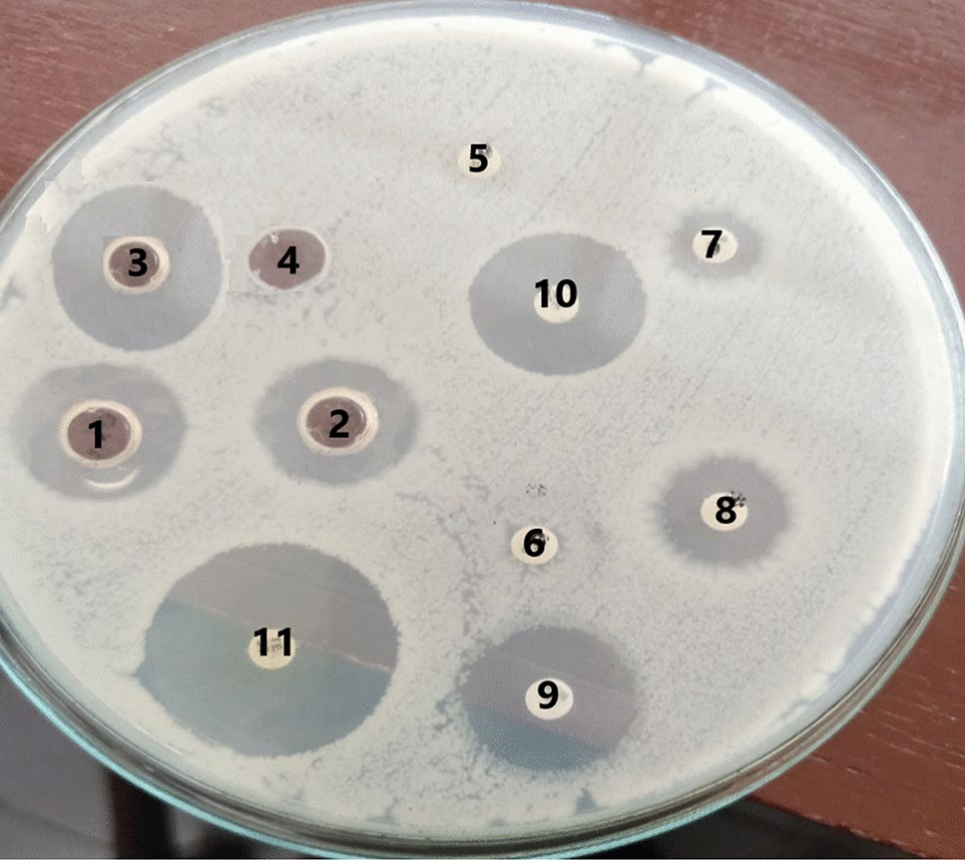
Antibacterial activity of EOO (1), CFF (2), and EOO/CFF (3) in the presence of water as a negative control (4) and some antibiotics including cefotaxime (5), oxacillin (6), penicillin (7), amoxicillin (8) vancomycin (9), gentamicin (10), and tetracycline (11).

The antibacterial activities of six different essential oils were tested against *B. cereus* RS1 by the well diffusion method. The results shown in Fig. 3 indicated that orange oil had significant inhibitory activity against the food pathogen *B. cereus,* with an inhibition zone of 19.00 ± 1.322 mm. No inhibition was detected after the addition of the other tested EOs. Additionally, the essential orange oil was tested in comparison to CFF and a mixture of CFF and EOO against *B. cereus* RS1, as shown in Fig. 4. There was highly significant inhibition in the case of EOO/CFF (22.30 ± 2.57 mm), followed by CFF (19.71 ± 2.93 mm). An antibiotic susceptibility test was performed to determine which drugs inhibited the growth of *B. cereus* RS1 in comparison with the experimental antibiofilm agents CFF, EOO, and EOO/CFF (Fig. 5). The results revealed that *B. cereus* RS1 was highly resistant to cefotaxime and oxacillin and susceptible to gentamicin (20.09 ± 1.934 mm), vancomycin (21.89 ± 2.14 mm), and tetracycline (25.28 ± 2.959 mm). Amoxicillin and penicillin had little inhibitory effect (10.13 ± 1.17 and 5.6 ± 1.29 mm, respectively) on *B. cereus* RS1. *B. cereus* RS1 was considered resistant to antibiotics of smaller-sized inhibition zones (amoxicillin and penicillin) according to the EUCAST guidelines.

### Determination of MIC values and antibiofilm activity of the CFF of *L. pentosus *RS2 and EOs against *B. cereus* RS1

The bacterial CFFs of *L. pentosus* RS2 and EOO were tested at different concentrations for their antibacterial activity against *B. cereus* RS1. A total of 400 μl ml^-1^ of CFF and 16 μl ml^-1^ of EOO completely inhibited the growth of *B. cereus* after 24 h of incubation at 37 °C, and the MIC values were recorded. Additionally, the effects of the cell-free filtrates of *L. pentosus,* EOO, and CFF/EOO on the growth of *B. cereus* were tested. The results revealed highly significant inhibition of biofilm formation by *B. cereus* RS1 after treatment with the EOO/CFF mixture (*O.D.* 0.363) compared with treatment with each agent alone. The antibacterial activity of CFF was greater than that of EOO, whereas the difference in inhibitory activity between EOO and CFF was not significant (*P* > 0.05).

The EOO/CFF mixture was examined for its antibiofilm activity against *B. cereus* RS1 by the crystal violet assay method via the coincubation method. The results revealed significant inhibition of biofilm formation by *B. cereus* (*P* < 0.05) after treatment with all the tested materials. Assaying via the crystal violet method, via the coincubation method revealed that the CFF/EOO mixture produced a greater antibiofilm effect against *B. cereus,* with an adherence of 20%, than did CFF and EOO.

### Anti-adherence activities of the CFF of LAB, orange oil and CFF/EOO against biofilm production by *B. cereus* RS1 on commercial area

**Fig. 6:**
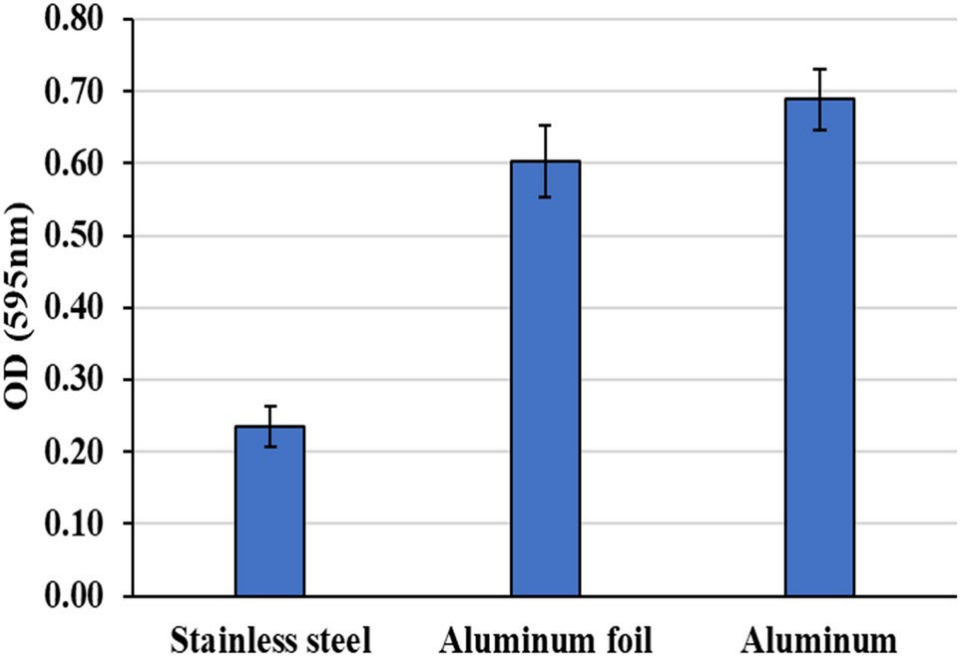
Biofilm formation of *B. cereus* RS1 on commercial surfaces. Data were represented as mean ± standard deviation (SD). The mean was calculated based on data obtained from triplicate independent measurements.

The adhesion of *B. cereus* RS1 on three important commercial surfaces, stainless steel, aluminum, and aluminum foil, was tested. The results in Fig. 6 show that the highest biofilm production was observed after 48 h of incubation of *B. cereus* RS1 on the aluminum surface (O.D. 0.689 ± 0.042), followed by the aluminum foil surface (O.D. 0.603 ± 0.049)*,* where the lowest biofilm formation was observed on the stainless-steel surface (O.D. 0.235 ± 0.028). The application of antibiofilm agents revealed that CFF, EOO and CFF/EOO inhibited biofilm formation on all the tested surfaces. The results in Table 2 revealed that CFF/EOO was the most significant reduction in biofilm production, with adherence percentages of 33.3%, 36.3%, and 40.8% on stainless steel, aluminum foil, and aluminum, respectively, followed by CFF and EOO.

**Table 2:** Anti-biofilm activity of the CFF of *L. pentosus* RS2, EOO, and CFF/EOO mix against the adherence of *B. cereus* RS1 to different commercial food surfaces via the crystal violet assay method.

Surface type	Control	CFF		EOO		Mix of CFF/EOO	
		Sub-MIC	%adherence	Sub-MIC	% adherence	½ Sub-MIC of CFS and ½ sub-MIC of orange oil	%adherence
Stainless steel	0.267 ± 0.055^a,c^	0.125 ± 0.014^a,c,d^	46.8	0.139 ± 0.018^a,c,e^	52.1	0.089 ± 0.019^a,c^	33.3
Aluminum foil	0.603 ± 0.096^a,b,c^	0.317 ± 0.036^a,b,c,d^	52.6	0.335 ± 0.047^a,b,c,e^	55.6	0.219 ± 0.025^a,b,c^	36.3
Aluminum	0.689 ± 0.088^a,c^	0.411 ± 0.033^a,b,c,d^	59.7	0.450 ± 0.051^a,b,c,e^	65.3	0.281 ± 0.048^a,c^	40.8

The data are presented as the means ± standard deviations (SDs).

Different letters (a–e) in the same column indicate statistically significant differences at *P* < 0.05 according to one-way ANOVA followed by Tukey’s HSD test for pairwise comparisons among different treatments.

### Molecular analysis of essential regulatory genes involved in biofilm formation

#### PCR and sequencing analysis

**Fig. 7:**
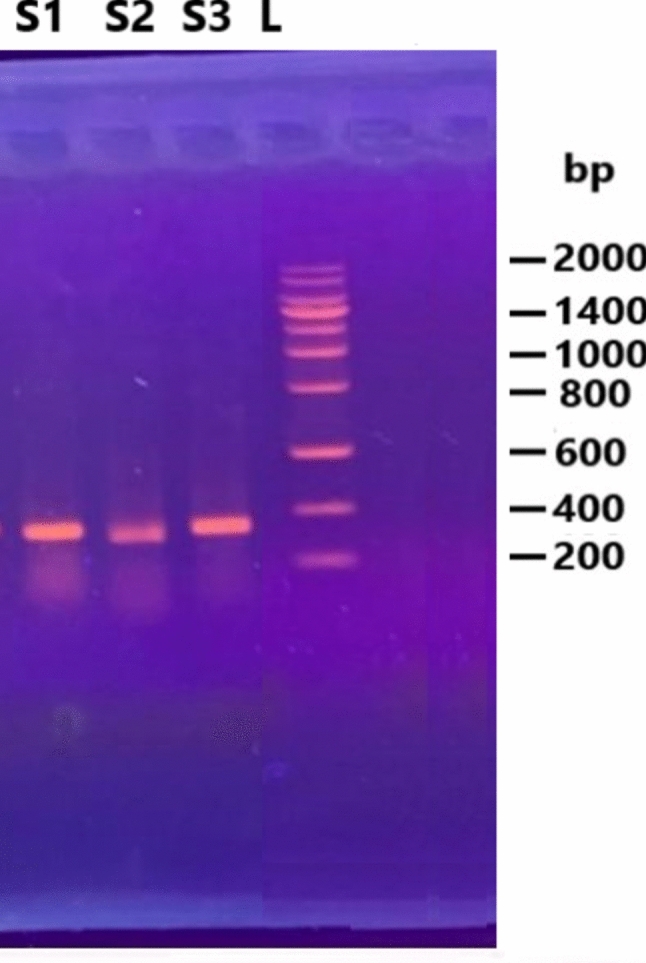
Gel electrophoresis image of q-rtPCR product of biofilm biosynthesizing genes, *sinR* (S1), *calY* (S2), and *spo0A* (S3) amplified from cDNA of untreated of *B. cereus* strain. L referred to 200 bp ladder DNA strand.

**Fig. 8:**
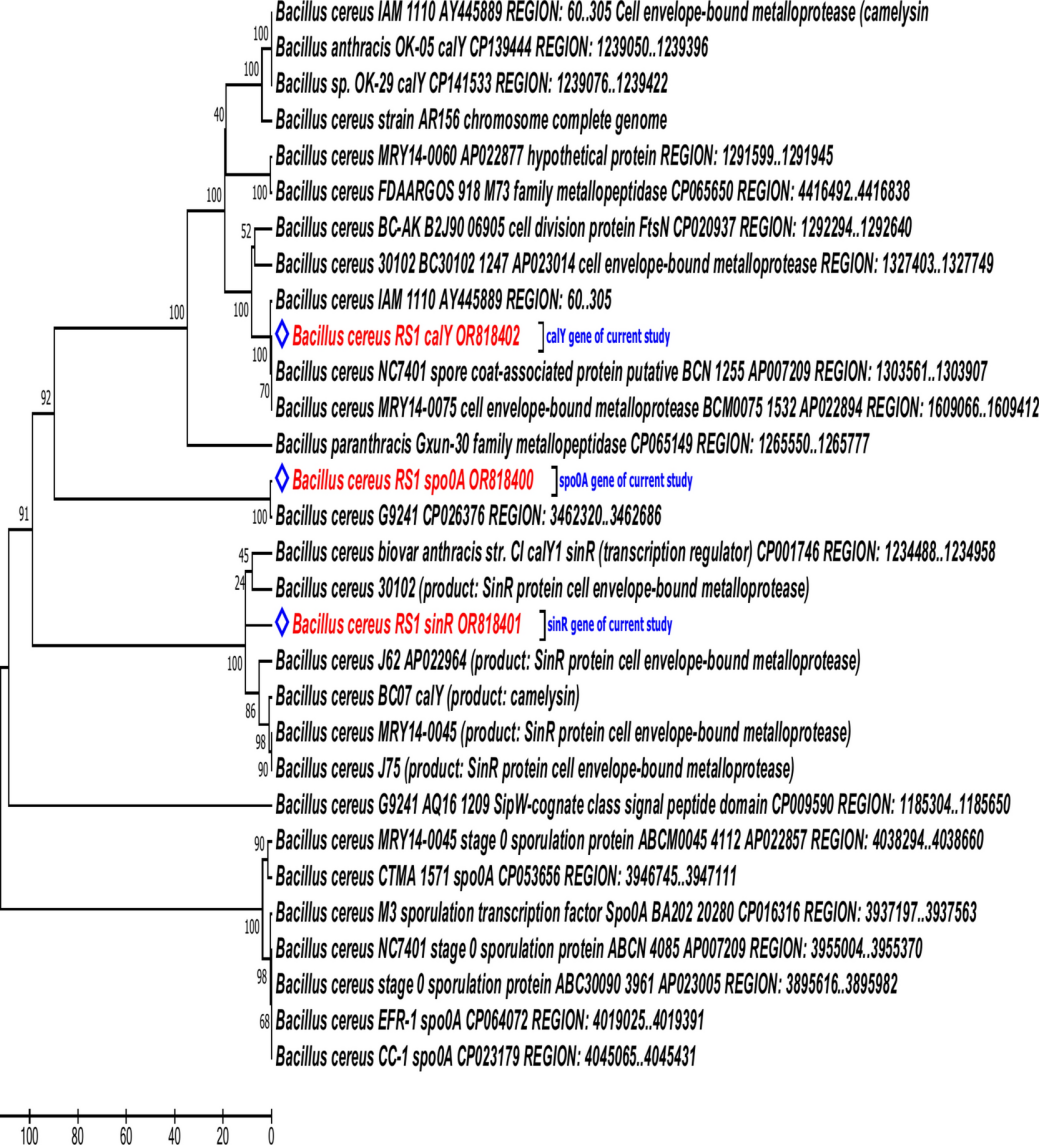
Phylogenetic analysis of *spo0A, sinR, calY* genes responsible for biofilm biosynthesis in *B. cereus* showing their relationship with each other and with closely related gene of different bacterial strains of the same species retrieved from NCBI GenBank. The evolutionary history was inferred using the Maximum Parsimony method. Tree #1 out of 2 most parsimonious trees (length = 37) is shown. The consistency index is (0.645161), the retention index is (0.780000), and the composite index is 0.548108 (0.503226) for all sites and parsimony-informative sites (in parentheses). The percentage of replicate trees in which the associated taxa clustered together in the bootstrap test (1000 replicates) are shown next to the branches. The MP tree was obtained using the Subtree-Pruning-Regrafting (SPR) algorithm with search level 1 in which the initial trees were obtained by the random addition of sequences (10 replicates). The analysis involved 11 nucleotide sequences. Codon positions included were 1st + 2nd + 3rd. All positions containing gaps and missing data were eliminated. There was a total of 324 positions in the final dataset. Evolutionary analyses were conducted in MEGA11software.

**Fig. 9:**
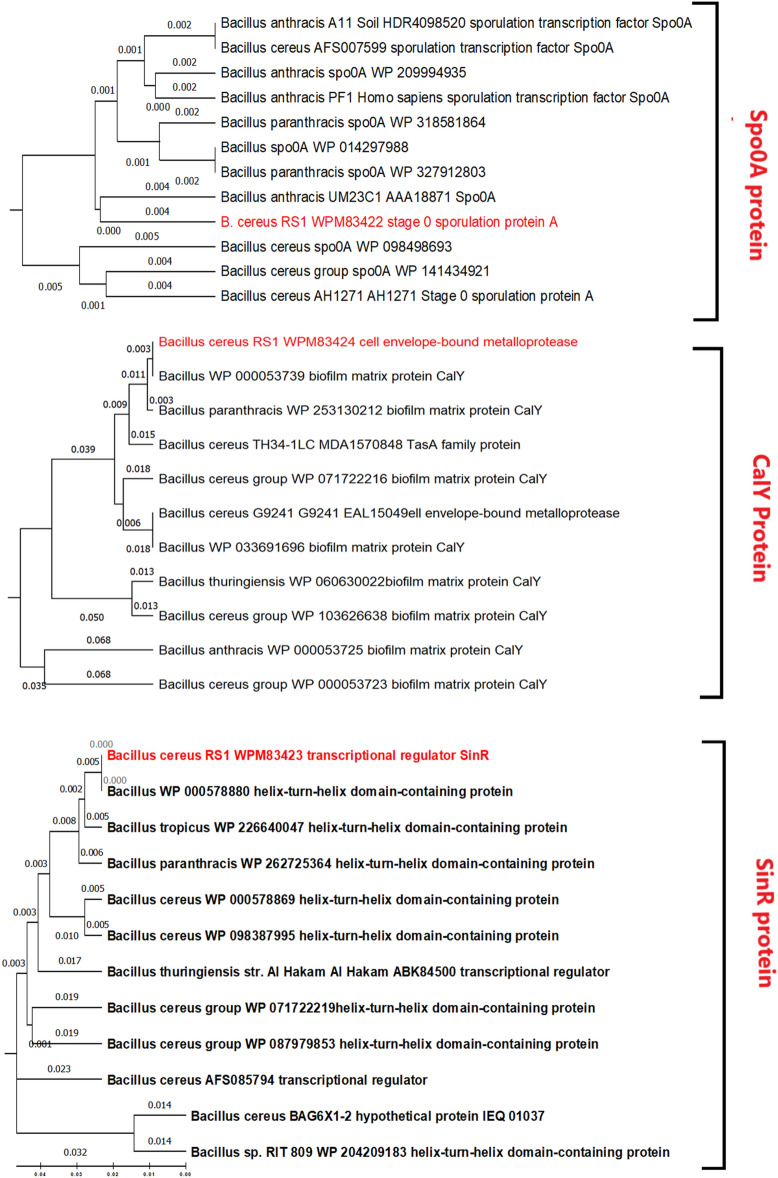
Phylogenetic analysis of Spo0A, SinR, CalY protein of biofilm biosynthesis in *B. cereus* RS1 showing their relationship with the closely related protein of different bacterial strains retrieved from NCBI GenBank. The evolutionary history was inferred using the Maximum Parsimony method. The percentage of replicate trees in which the associated taxa clustered together in the bootstrap test (1000 replicates) are shown next to the branches. Evolutionary analyses were conducted in MEGA11software.

**Fig. 10:**
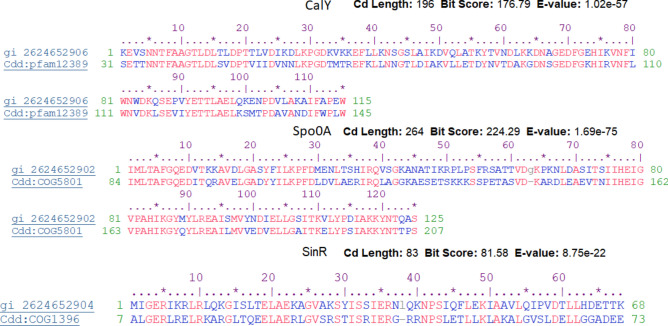
A conserved region of putative protein of biofilm forming genes with respective protein family. gi: our amino acid sequences of biofilm forming genes, cdd: conserved domain of protein family.

PCR and gel electrophoresis analyses of three biofilm regulatory genes, namely, *calY, spo0A*, and *sinR*, revealed strong amplification bands in *B. cereus* RS1 (Fig. 7). The amplified DNA fragments of the biofilm coding genes were sequenced in the two directions. The released sequences of all biofilm-coding genes were matched with the corresponding similar sequences registered in the GenBank database. The analysis of the biofilm regulatory genes detected that the obtained sequences presented 98–99% identity to the similar coding genes of *Bacillus* species recorded in the GenBank databases. The obtained sequences of *spo0A, sinR,* and *calY* genes in *B. cereus* RS1 were submitted at the GenBank database with the accession numbers OR818400.1, OR818401.1, and OR818402.1, respectively. Full annotation of the biofilm coding genes was recorded in Table 3. Phylogenetic analysis of the biofilm coding genes revealed a highly significant relationship between the three analyzed genes and other related genes recorded in the GenBank database (Fig. 8). The putative proteins of all the sequenced genes also presented high identity with the orthologous proteins of biofilm-coding genes in *Bacillus* species (Fig. 9). The conserved regions of biofilm homologous protein present in other strains are shown in Fig. 10.

**Table 3:** Annotation of the biofilm coding genes *calY, spo0A*, and *sinR* in *B. cereus* genome.

Gene symbol	Gene accession number	Locus tag	Gene description	Protein accession number
*calY*	OR818402.1	FORC47_RS06660	Biofilm matrix protein CalY (similar to *tasA*)	WPM83424.1
*spo0A*	OR818400.1	FORC47_RS21540	Sporulation transcription factor Spo0A	WPM83422
*sinR*	OR818401.1	FORC47_1178	SinR regulator of post-exponential-phase	WPM83423

#### Expression analysis of the biofilm-coding genes

**Fig. 11:**
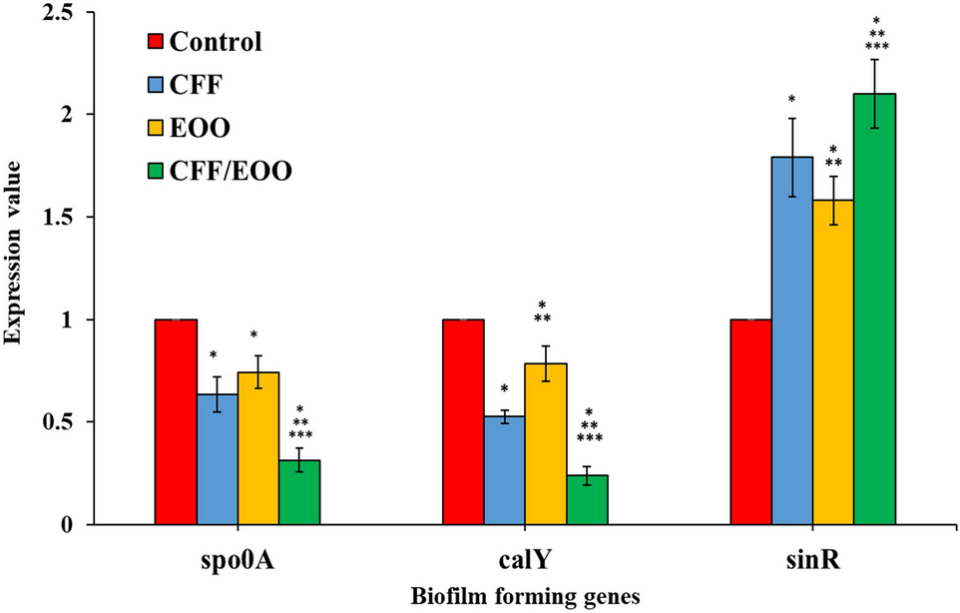
Fold change of biofilm forming genes (*sinR, calY*, and *spo0A*) of *B. cereus* RS1 treated with CFF, EOO, and EOO and CFF/EOO mix compared to untreated sample (control) after 17 h of incubation at 37 °C. The error bars and asterisks (*) represent standard deviations and significant differences (*p* < 0.05), respectively. *indicate statistically significant differences at *P* < 0.05 when comparing control vs. other treatments. **indicate statistically significant differences at *P* < 0.05 when comparing CFF vs. EOO and mix CFF/EOO. ***indicate statistically significant differences at *P* < 0.05 when comparing EOO vs. CFF/EOO mix.

The transcription value of the biofilm-coding genes (*calY* and *spo0A*) and the negative regulator gene (*sinR*) was evaluated in the strong biofilm-producing strain of *B. cereus* RS1 via q-RTPCR analysis. The fold change of the three genes was calculated in *B. cereus* after and before treatment with the EOO, CFF, and EOO/CFF mixtures. The results are recorded in Table 4 and Fig. 11. The transcription values of *spo0A*, *calY* and *sinR* were 0.6329 ± 0.085, 0.5249 ± 0.0321, and 1.79005, respectively, in the case of treatment with CFF; 0.7423 ± 0.079, 0.7846 ± 0.0849, and 1.580083, respectively, in the case of treatment with EOO; and 0.3143 ± 0.058, 0.2382 ± 0.045, and 2.099433, respectively, in the case of treatment with the EOO/CFF mixture. Compared with that of the untreated bacteria, the expression value of both the *calY* and *spo0A* was downregulated whereas the *sinR* gene was upregulated in all the treatments. The expression values of both *calY* and *spo0A* genes were highly significantly lower in the cells treated with EOO/CFF than in those treated with CFF or EOO alone. On the other hand, the expression of the biofilm regulator gene *sinR* was highly significantly greater in all the treated samples than in the control samples. The expression of the negative regulatory gene *sinR* was highly significantly greater in the cells treated with EOO/CFF than in those treated with CFF or EOO alone. The expression value of *sinR* was twofold greater than that of the control sample in the case of treatment with CFF/EOO (*p* < 0.0001).

**Table 4:** Expression analysis of biofilm-coding genes (*spo0A*, *calY*, and *sinR*) of *B. cereus* before (control) and after treatment with CFF, EOO, and a mixture of EOO and CFF. The *dnsJ* represents the housekeeping gene.

Treatment	*dnaJ*	*spo0A*		*calY*		*sinR*	
	CT	CT	Fold change	CT	Fold change	CT	Fold change
Control	19.58	21.17	–	22.63	–	25.19	–
CFF	19.42	21.67	0.633 ± 0.085^a,b^	23.40	0.5249 ± 0.0321^a,b^	24.19	1.790 ± 0.19^a,b^
EOO	20.33	22.35	0.742 ± 0.079^a,c^	23.73	0.7846 ± 0.0849^a,b,c^	25.28	1.580 ± 0.118^a,b,c^
CFF&EOO	19.82	23.08	0.314 ± 0.058^a,b,c^	24.94	0.238 ± 0.045^a,b,c^	24.36	2.099 ± 0.167^a,b,c^

The data are presented as the means ± standard deviations (SDs).

Different letters (a–c) in the same column indicate statistically significant differences at *P* < 0.05 according to one-way ANOVA followed by Tukey’s HSD test for pairwise comparisons among different treatments.

^a^Comparison between the control and other treatments.

^b^Comparison between CFF and EOO. and mixed CFF&EOO.

^c^Comparison between EOO and mixed CFF&EOO.

## Discussion

The production of biofilms on food tools can result in major hygienic issues, including an increase in foodborne diseases and economic losses resulting from food spoilage. Traditional cleaning substances may contain chemical detergents and other surfactants, which can cause various health problems when the equipment is not fully rinsed. Therefore, the development of natural antibacterial and antibiofilm agents to address this problem is urgently needed. The present study focused on isolating foodborne pathogens, aiming at the species *B. cereus,* and investigating a new strategy to inhibit bacterial contamination and biofilm formation via natural products. Additionally, the expression of three important genes involved in biofilm formation in *B. cereus* was studied under the effects of natural antibiofilm agents. Forty bacterial pathogens were isolated from 10 types of Egyptian food. It was observed that Gram-negative bacteria were more common than Gram-positive bacteria in all the tested sources, where 90% of all the isolated bacteria were characterized as Gram-negative and only 10% were characterized as Gram-positive bacteria, the most common isolates belonging to the Enterobacteriaceae. This result may be due to the ability of Enterobacteriaceae to grow in simple broth media, as they can ferment a broad variety of carbohydrates^48^. *B. cereus* was the strongest strain for biofilm formation under the present experimental conditions. *B. cereus* can be used to synthesize biofilms in different beverages and food industries. Formation of bacterial biofilms in food products can cause foodborne infections^49^. Biofilm synthesis by foodborne pathogen plays a significant role in the pathophysiology of various diseases in animals and humans^50^. Biofilms increase the resistance of bacterial pathogens to antibiotics, 1000-fold more than do planktonic bacteria. In our research, the inhibition of biofilm production via LAB and EOO was investigated*.* The results revealed that *L. pentosus* had the greatest inhibitory impact on *B. cereus* growth and biofilm synthesizing. The antimicrobial activity of the *L. pentosus* filtrate may be due to the production of inhibitory bioactive molecules that inhibit *B. cereus* growth and significantly decrease biofilm formation. A previous study reported the production of several metabolic molecules, such as hydrogen peroxide, diacetyl, carbon dioxide, bacteriocin, ethanol, and organic acids, by lactic acid bacteria (LAB), and these metabolic molecules have antibacterial activity against foodborne pathogens^51^. Pathogenic bacteria are naturally sensitive to acidic conditions and can be destroyed in low-acidity cultures ^52^. Additionally, Amenu and Bacha^53^ reported that the bioactive metabolite of LAB has significant effects against food spoilage bacteria. Mokoena^54^ reported that some LAB, with probiotic functions and GRAS (generally regarded as safe) status, are applied in the pharmaceutical and food industries. This group of bacteria is an effective agent for inhibiting pathogenic foodborne bacteria^55^. In the food industry, foodborne pathogens can produce biofilms, which can lead to food spoilage, harming the health of consumers^56^. Additionally, a survey study on the inhibitory effects of essential oils revealed that orange oil had strong antibacterial and antibiofilm activities against *B. cereus*. Similarly, Kamel et al.^57^ reported that citrus oil contains active biomolecules, such as antioxidants and anticancer agents, which have antimicrobial activities against various pathogens. Essential oils are important antimicrobial and antibiofilm agents because they contain various active ingredients^21^. They are vigorous agents that decrease antimicrobial resistance^58^ due to their considerable therapeutic properties, such as antioxidants, antibacterial, and antiseptic activities^19,20^. EOs from citrus were previously reported by O’bryan et al.^59^ as excellent antibacterial agents against foodborne pathogens. The biological efficiency of citrus EOs as antimicrobial and antioxidant agents, among others, has been used previously for controlling foodborne pathogens^60^. The effect of the plant essential oil on biofilm formation by pathogenic bacteria was also investigated by Emad et al.^61^. They reported that neem oil has promising inhibitory effects on bacterial growth and biofilm formation by *Staphylococcus aureus* and *Pseudomonas aeruginosa.* Abed et al.^62^ found strong antibacterial activity of rosemary volatile oil against *B. cereus* isolated from some canned meat products. The present study was extended to investigate the inhibitory effects of the EOO/CFF mixture on the growth of *B. cereus* and biofilm formation in comparison with each agent alone. The results of the MIC test revealed a highly significant inhibition of bacterial growth and biofilm formation by *B. cereus* RS1 after treatment with the EOO, CFF, and EOO/CFF mixture. The results indicated that the EOO/CFF mixture was the strongest agent against bacterial growth and biofilm formation, followed by CFF and EOO. This result may be due to the synergistic effect of the bioactive compounds present in CFF with the active ingredients of EOO. Both compounds have antimicrobial activity, and their combination increased their inhibitory effects on *B. cereus* growth and decreased biofilm formation. This finding agrees with Fawzi and Ahmed et al.^63^**,** who indicated that combining various antimicrobial agents with different modes of action resulted in increased potency and a wide range of inhibition, including multidrug-resistant bacteria. Additionally, the antibiofilm activity of EOO and CFF/EOO against the adherence of *B. cereus* RS1 was tested on the surfaces of food processing contact materials, stainless steel, aluminum, and aluminum foil. The results revealed that CFF, EOO, and CFF/EOO inhibited biofilm formation by *B. cereus* on all the tested surfaces. CFF/EOO resulted in the most significant reduction in biofilm formation on all surfaces. Moreover, the inhibitory activity of CFF/EOO against *B. cereus* RS1 was better on stainless steel than on the other materials. The inhibitory effect of biofilm production on surfaces may be owing to marking molecules that prevent the binding of bacterial cells to layers, stopping the biosynthesis of polymers in the extracellular products that control linkage between bacteria. We confirmed the inhibitory effects of the experimental antibiofilm agents by analyzing the expression of three virulence genes involved in biofilm formation, *sinR, calY,* and *spo0A*. The results of this study revealed that the CFF, EOO, and CFF/EOO mixtures downregulated the expression of *calY* and *spo0A* and upregulated the expression of *sinR*. The CFF/EOO mixture was more effective at suppressing *calY* and *spo0A* and activating the negative regulator gene *sinR*. These results were consistent with the experimental analyses of biofilm inhibition, which suggested that the suppression of both the *calY* and spo0A genes was a significant reason that natural products inhibited biofilm formation in *B. cereus*. However, these products significantly induced the expression of *sinR* and suppressed both *calY* and *spo0A.* The *sinR* gene is a negative regulator of virulence genes involved in biofilm formation. The master regulator protein, SinR, regulates the transcription of three different gene operons, tasA-yqxM-sipW, which are responsible for the biofilms-formation. SinR joins multiple sites in the promoter region of the operon and represses their transcription. SinR controls the transcription of approximately 18 genes responsible for developing an extracellular matrix, that permits the adherence of the cells to each other in biofilms^28^. SinR protein is a transcriptional regulator for the exopolysaccharide *epsA-O* operon ^27^. This operon is required for the biosynthesis of the extracellular matrix that rolls a chain of cells through biofilm development, and its transcription is directly under the negative control of the DNA-binding protein^27^. CalY protein was also investigated in *B. cereus* as a cell-surface protein that can bind to mucin and fibronectin^64^. Thus, CalY is considered one of the components of the biofilm matrix. The *calY* gene results from gene duplication of the *tasA* gene, a process that can lead to the evolution of moonlighting proteins^65^. Candela et al.^65^ suggested that CalY could be a bifunctional protein involved in both biofilm matrix fabrication and adhesion to host tissues. They reported that CalY is a vital virulent factor and an essential component of biofilms in *B. thuringiensis*. The *AbrB* gene is a transcription factor that negatively regulates biofilm production in *B. subtilis*. Thus, AbrB protein may inhibit the transcription of genes participating in intercellular adhesion. Spo0A represses the transcription of the *abrB* gene through its binding to the promoter region^66^. Thus, the main role of *spo0A* in biofilm biosynthesis is to negatively regulate the transcription of the *abrB* gene; consequently, *spo0A* activates biofilm formation^67^. Although the transcription factor Spo0A is required for sporulation, some signals that regulate sporulation may also regulate biofilm formation^67^. Owing to the crucial role of these genes, *sinR, calY*, and *spo0A*, in biofilm formation, we focused on analyzing their expression under the effects of biofilm inhibitor agents. This study is the first report concerning the application of bacterial CFF and EOO as inhibitory agents against bacterial growth and biofilm formation and to analyze the expression values of three important genes, *sinR, calY*, and *spo0A*, that are involved in biofilm biosynthesis and regulation in the foodborne pathogen *B. cereus* RS1.

## Conclusion

It was concluded from this study that the Gram-negative bacteria were more distributed than the Gram-positive bacteria in different Egyptian food samples. The *B. cereus* RS1 was the most potent biofilm-producer pathogen. The essential orange oil and cell-free filtrate of *L. pentosus* RS2 had significant inhibitory activity against the food pathogen, *B*. *cereus* RS1. The CFF/EOO mix could inhibit biofilm formation by food pathogens on commercial surfaces. Moreover, the biofilm regulatory genes, *sinR*, *calY*, and *spo0A*, were detected in *B. cereus* RS1 genome and their expression was significantly affected by the antibiofilm agents. There was a strong relation between the expression analysis of biofilm-coding genes and the physiological test of all inhibitory agents. These results encourage the application of EOO/CFF as antimicrobial and antibiofilm agents against the food-pathogenic bacteria, *B. cereus*.

## Data Availability

All authors stated that all the data are available in the manuscript.

## Code availability

Data is provided within the manuscript.
